# *CCDC122-LACC1* gene polymorphism is associated with protection against leprosy in a population from Northeastern Brazil: a case-control study

**DOI:** 10.1186/s12879-025-12391-3

**Published:** 2025-12-22

**Authors:** Heloisa de Almeida Freitas, Karla Regina Celestino Nogueira, Allan Ribeiro Reis Scharf Costa, Luana Karen Correia dos Santos, Ana Tércia Paulo Silva, Susana Paiva Oliveira, Vanderson Ferreira Lima, Nathalí da Silva Araújo, Walcelia Oliveira dos Santos, Poliana Pinheiro Pascoal, José Karlison Tavares Valeriano, Carlos Alberto de Carvalho Fraga, Carlos Dornels Freire de Souza, Emiliano de Oliveira Barreto, Elaine Virgínia Martins de Souza Figueiredo, Rodrigo Feliciano do Carmo, Carolinne Sales-Marques

**Affiliations:** 1 Postgraduate Program in Health Sciences, Institute of Biological and Health Sciences, Federal University of Alagoas, Maceió, Alagoas Brazil; 2 Oswaldo Cruz Institute, Oswaldo Cruz Foundation, Rio de Janeiro, Rio de Janeiro, Brasil; 3 Postgraduate Program in Health and Biological Sciences, Federal University of the São Francisco Valley, Petrolina, Pernambuco Brasil; 4 Federal University of Alagoas, Arapiraca, Alagoas Brasil; 5 Arapiraca Integrated Reference Center, Arapiraca, Alagoas Brazil

**Keywords:** Medical and human genetics, SNPs, Immunology, Case-control, Brazilian population

## Abstract

**Introduction:**

Leprosy is a disease caused by *Mycobacterium leprae* and *Mycobacterium lepromatosis*, affecting the skin and peripheral nerves. In 2022, Brazil registered more than 19,000 new cases, considered a public health problem in the country. Interactions between the pathogen, host genetics, and the environment are factors to be considered for the development of leprosy. The *CCDC122-LACC1* and *IL23R* genes play important roles in immune regulation. To understand the genetic basis of leprosy, this study aimed to investigate the association of SNPs belonging to the *CCDC122-LACC1* and *IL23R* genes with leprosy in a population from northeastern Brazil.

**Methods:**

A case-control study was conducted, using confirmed leprosy patients (cases) and individuals health donors (controls). Subjects were recruited from Northeastern states of Brazil, with a total sample size 952 (562 in Alagoas and 390 in Bahia and Pernambuco, being 488 samples from cases (298 in Alagoas and 190 in Bahia and Pernambuco) and 464 controls (264 in Alagoas and 200 in Bahia and Pernambuco). Genotyping of the SNPs *CCDC122-LACC1* and *IL23R* was performed using real-time PCR (Taqman, StepOne Plus™). Associations were quantified using odds ratios with 95% confidence intervals, and were performed in the R environment (v.3.4.4).

**Results:**

The results demonstrated that SNP *CDC122-LACC1* was associated with protection against leprosy in the population of Alagoas (OR_CC_ = 0.58, *p* = 0.02), and in the combined analysis of the populations of Northeastern Brazil (OR_CC_ = 0.65, *p* = 0.02), which was also associated with the multibacillary operational classification in the populations mentioned above. While analyzing the *IL23R* polymorphism, no association with leprosy was observed in any of the analyses performed in the study populations, and no association was identified with the operational classifications of the disease.

**Conclusions:**

The SNP rs4942254 in the *CCDC122-LACC1* gene was associated with protection against development of leprosy.

**Supplementary Information:**

The online version contains supplementary material available at 10.1186/s12879-025-12391-3.

## Background

Leprosy is a chronic, infectious and ancient granulomatous disease, with a wide spectrum of clinical manifestations. Its etiological agent is the *Mycobacterium leprae* (*M. leprae*), an acid-fast alcohol bacillus with low genetic evolution. Recently, *Mycobacterium lepromatosis* (*M. lepromatosis*) was also identified as the cause of leprosy [1]. These bacilli affect skin macrophages and the peripheral nervous system, mainly Schwann cells, which can lead to serious neuromotor and autonomic nerve damage, and consequently, leading to disabilities and physical deformities such as muscular atrophy that can become irreversible [2, 3].

Even though it is treatable, leprosy remains one of the main causes of disability and social stigma, with more than 200,000 new cases diagnosed worldwide each year [4, 5]. The disease is still considered a public health problem in many low- and middle-income countries, such as Brazil. In 2022, the country recorded 19,635 new cases of the disease, with a detection rate of new cases of 9.67 per 100,000 inhabitants [6].

The long incubation period and insidious symptoms and signs of leprosy can make diagnosis difficult and, consequently, delay treatment. Exposed individuals present a wide diversity of symptoms reflecting interactions between the host’s immune response and the bacteria. Susceptible individuals can be affected along a spectrum of severity depending on the state of the immune system, with greater resistance occurring in tuberculoid leprosy and decreasing through the lepromatous form that manifests in those with lower resistance [7–9].

Factors to be considered for the development of leprosy are characteristics of the pathogen, the genetics of the host and the environment that will act. Due to this, only a small proportion of individuals develop the clinically evident disease [10, 11]. Among the host’s genetic context, there are immune response genes, and within them, single or simple nucleotide polymorphisms (SNPs), which are genetic markers that may be associated with risk or protection for the development of leprosy [12, 13].

To understand the genetic basis of susceptibility to leprosy, association analyses were performed based on candidate genes, such as the *CCDC122-LACC1* gene responsible for increasing the response induced by innate receptors, regulating the bioenergetic state of macrophages, metabolic function and secretion of cytokines. This gene is also involved in the process of cell signaling, bacterial elimination, inflammation and tissue injury [14, 15]. The gene is expressed in the cytoplasm and mitochondria, and after stimulation of macrophages by *NOD2*, the CCDC122-LACC1 protein molecule associates with the signaling complex that is critical for optimal NOD2-induced signaling, cytokine secretion and bacterial elimination. However, to date, there are no studies that explain the mechanisms that are related to its biological effects on leprosy [15].

The *IL23R* gene is responsible for the production of the protein called interleukin 23 receptor (IL-23), and plays an important role in immune regulation, being incorporated into the outer membrane of several types of immune system cells, such as: T cells, natural killer (NK), monocytes and dendritic cells [16–18]. The *IL23R* gene has been shown to be a candidate gene for association with leprosy, since SNPs in this gene have been associated with increased susceptibility to the disease, also affecting the profile of inflammatory cytokines in human cells [19].

Studies have shown that *IL23R* protein levels are altered upon infection by *M. leprae*, suggesting that this molecule is involved in defense mechanisms against the pathogen [20]. The protein encoded by *IL23R* forms the receptor for the cytokine interleukin (IL)-23 that is part of a signaling pathway involving the gene product of another leprosy-associated locus, *TNFSF15*. Together with the IL-12 β1 subunit (encoded by *IL12RB1*), *IL23R* is a part of the IL-12, IL-23, and IFN-γ cascades, which have been suggested to play essential roles in immunity to mycobacteria [21]. The SNP rs3762318 is located within a linkage disequilibrium in the 150 kb block where *IL23R* and *C1orf141* are present [16].

The aim of this study was to investigate whether polymorphisms at *CCDC122-LACC1* and *IL23R* genes have genetic influences on the development of leprosy in two populations from Northeastern Brazil. We expect that this gene candidate investigation in the studied population may help in the understanding of the pathophysiology of leprosy and its underlying mechanisms, contributing to the comprehension of the immunological mechanisms involved in susceptibility to the disease, including the function of related genes, immune response, cell signaling and inflammation. The aim of this study was to analyze the association of SNPs in the *CCDC122-LACC1* and *IL23R* genes with leprosy in a population sample from Northeastern Brazil.

## Methods

### Ethical aspects

The individuals who agreed to participate in the research were presented to the research and signed the FICF (Free and Informed Consent Form). The project was submitted and approved by the Ethics Committee of the Federal University of Alagoas (UFAL) under protocol number: 4.439.041 (CAAE: 57828716.0.0000.5013), and protocol number: 068649/2016. It was also authorized by the Ethics and Research Committee of the Hospital of Clinics of the Federal University of Pernambuco (HC/UFPE) (CAAE: 66179617.7.0000.5196).

### Subjects and study design

**Fig. 1:**
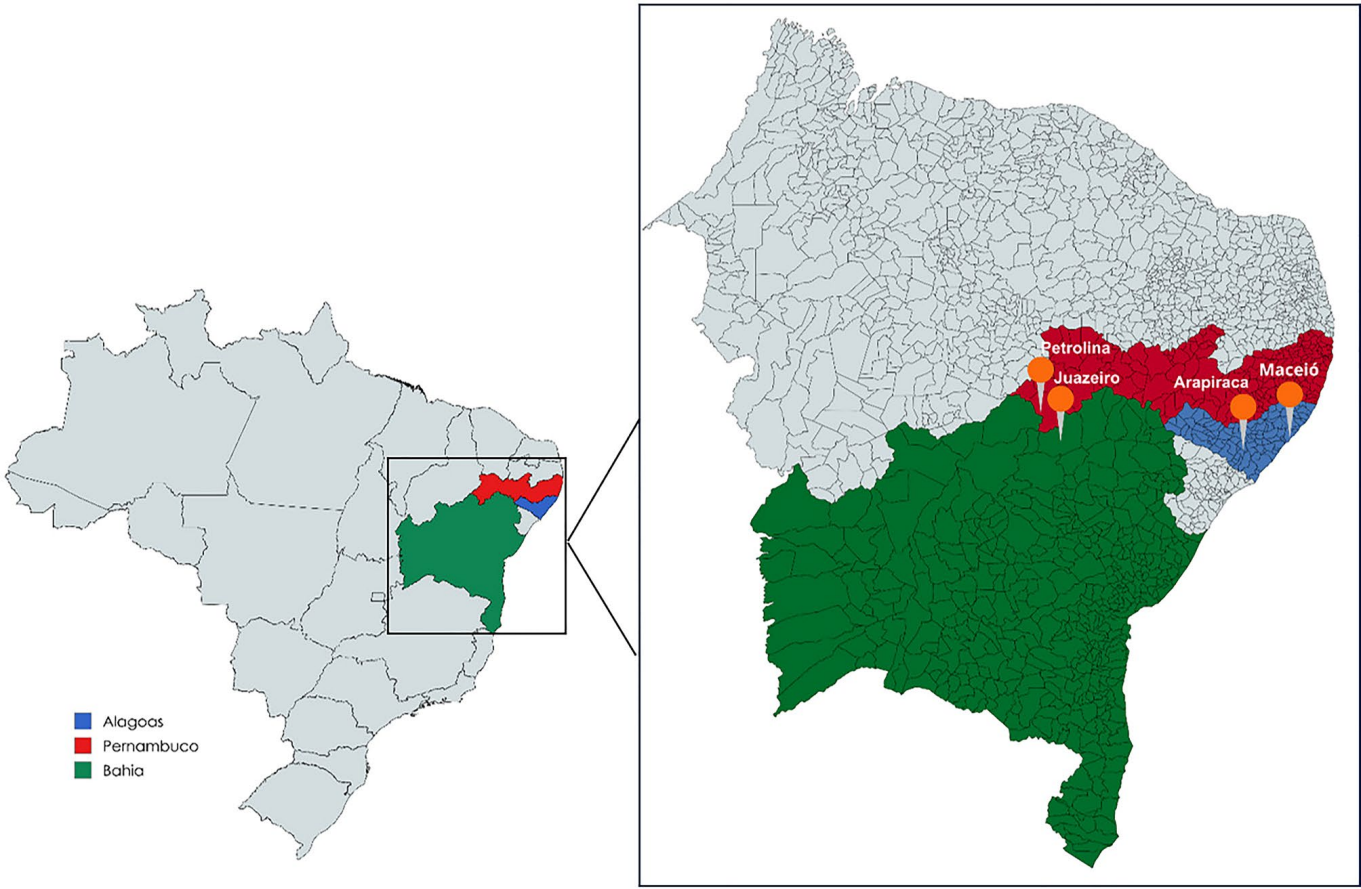
Geographic distribution of participating study populations in Northeastern Brazil. The participating states are highlighted in different colors: Alagoas in blue, Pernambuco in red and Bahia in green. The gray area does not contain populations participating in the study. The head pins represent the cities, where the recruitment and collection centers for cases and controls are located. Source: Research data, 2024

The aim of this study was to analyze the association of SNPs in the *CCDC122-LACC1* and *IL23R* genes with leprosy in a population sample from Northeastern Brazil. The present study is a retrospective case-control genetic association study carried out with a population sample from the state of Alagoas (between coordinates 9°08’ S − 10°12’ S and 35°30’ W − 37°45’ W; Datum: SIRGAS2000) [22], and replicated in a sample from the states of (between coordinates 9°23’ 34” S − 40°30’ 28” W; Datum: SIRGAS2000) [22] and Bahia (between coordinates 9°24’ 50” S − 40°30’ 10” W; Datum: SIRGAS2000) [22], Northeastern Brazil (Fig. 1). The research was conducted by the Federal University of Alagoas (UFAL) and the Federal University of São Francisco Valley (UNIVASF). To compose the case group, patients with leprosy from 2018 to 2023 were recruited, in a non-probabilistic way, at the Integrated Reference Center of Arapiraca (CRIA) in Arapiraca, and at the Dr. Diógenes Jucá Bernardes (Center II) and Roland Simon (PAM vergel) health units in Maceió, State of Alagoas. In the state of Bahia, at the Dr. Altino Lemos Health Center in Juazeiro, and at the Petrolina Infectious Diseases Service in Petrolina, state of Pernambuco (Fig. 1). Related patients, pregnant women, and HIV carriers were excluded from the study. The sociodemographic and clinical information from each patient’s medical records was compiled and organized into an Excel spreadsheet using Microsoft Office 2016 (Microsoft Corp., Redmond, WA, USA). After collecting the information, all patients were distributed according to their operational classification, following the classification criteria suggested by the WHO [23], being paucibacillary (PB) - cases with up to five skin lesions and negative bacilloscopy- and multibacillary (MB) - cases with six or more lesions and/or positive bacilloscopy, allowing the standardization of clinical information of study. Controls were invited to participate in the study voluntarily at the routine blood donation centers of the Alagoas Blood Donation Center (HEMOAL), in Arapiraca, and the Bahia Blood Donation Center (HEMOBA), in Juazeiro, from 2018 to 2019. The individuals included in the study to compose the control group were those who reported no history of infectious diseases and were not relatives of any individual previously recruited for the experimental design.

### DNA extraction and quantification

DNA extraction from peripheral whole blood samples from patients and controls was performed using the Purelink™ Genomic DNA kit (Thermo Fisher Scientific, Carlsbad, CA, USA) in samples of Alagoas and ReliaPrep™ Blood gDNA Miniprep System (Promega, Madison, WI, EUA) in samples of Bahia and Pernambuco [24]. The quantity and quality of the extracted DNA was verified using the nucleic acid quantification method by spectrophotometry using NanoDrop™ One (Thermo Fisher Scientific, Carlsbad, CA, USA), by measuring the concentration/absorbance ratios and the parameters 280/260 nm (nanometer) and 260/230 nm, to infer the quality/purity of the DNA.

### Genotyping

From the DNA samples (10–50 ng/uL) the genotyping was performed by allelic discrimination through real-time PCR, using TaqMan-type assays (probes), and following the manufacturer’s protocol (Applied Biossystems, Waltham, MA, USA). The results were obtained using allelic discrimination software, which allows defining the genotypes of each sample. In this assay, specific fluorescent probes are used for each allele of the gene being investigated, and the characterization of the genotypes was based on the fluorescence intensity (StepOne Plus software). The SNPs of this study were selected based on genetic studies [14–18, 31, 34–36] and previous functional approaches listed in the LSHGD (Leprosy Susceptible Human Gene Database) database [25], related to leprosy and/or candidates of interest for investigation, using frequency criteria from the Ensembl database (https://www.ensembl.org/index.html), where it was possible to perceive the population allele frequencies of the chosen SNPs rs4942254 (*CCDC122-LACC1*). For the C allele the frequency was 42% and for the G allele rs3762318 (*IL23R*) it was 16%, in the American population. Their MAF (Minor Allele Frequency) frequencies > 5% indicated that they were suitable candidates for analysis in the population of Northeastern Brazil [26].

### Statistical analyses

A quantitative analysis was performed to compare the frequencies of genotyped SNPs between cases and controls. Combined and separate analyses were performed (population from Alagoas and populations from Pernambuco and Bahia). Initially, the frequencies of the genotypes were obtained and organized in a database. The χ2 test was performed to evaluate the allele, genotype and carrier frequencies of the two polymorphisms investigated in the study population to assess the Hardy-Weinberg Equilibrium (HWE). Subsequently, associations between SNPs and leprosy were quantified using odds ratios (OR) with 95% confidence intervals and corresponding p-values. The covariate sex was used in the analysis model, generating adjusted OR, CI and *p*-values. The analyses involving alleles, genotypes and carriers were performed in the R environment, using the “genetics” and “SNPassoc” (v.3.4.4) packages. To verify the reliability of the sample power, the GPower software (v.3.1) was used, based on the genotyping data using the statistical models “X² Tests - Goodness-of-fit Tests: Contingency Tables” and the type of power analysis was “post hoc”.

## Results

### Demographic and clinical characteristics of the studied population

The research used a total sample size of 952 (562 in Alagoas and 390 in Bahia and Pernambuco), with 488 samples from patients with leprosy (298 in Alagoas and 190 in Bahia and Pernambuco), and an average age of 49 years. Regarding gender, 61.9% (*n* = 302) were male and 38.1% (*n* = 186) were female. Regarding operational classification, 13.7% of patients were paucibacillary (PB) and 86.3% of patients were multibacillary (MB). In the control group, 464 healthy donor individuals were recruited (264 in Alagoas and 200 in Bahia and Pernambuco), with an average age of 34 years. A total of 61.9% (*n* = 287) were male and 38.1% (*n* = 177) were female. The sociodemographic and clinical information of the recruited populations are shown in more detail in Supplementary Table 1 (see Supplementary Table 1).

The results indicate that the population sample for all SNPs did not show deviation from the HWE (χ2 = 1.586; *p* = 0.21 for rs4942254 (*CCDC122-LACC1*) and χ2 = 0.145; *p* = 0.70 for rs3762318 (*IL23R*)). For the *CCDC122-LACC1* SNP, the power assessment reached 0.99 for the tested values, except for the *IL23R* SNP, which was lower (0.44). As for the isolated analysis of the population of Pernambuco and Bahia for the SNP *CCDC122-LACC1*, it did not present significant sample power (0.56), but the combination of the sample volume shows interest and greater statistical power (0.99), which justifies uniting the population of Northeastern Brazil into a single population. Due to the absence of statistically significant associations for comparing clinical forms (PB vs. MB), we did not include this power analysis. However, for the MB vs. Controls, sufficient sampling power was obtained for the *CCDC122-LACC1* SNP (0.99) and for the *IL23R* SNP the value obtained was lower (0.05).

### Association of polymorphisms in the *CCDC122-LACC1* and *IL23R* genes and leprosy per se

For the rs4942254 polymorphism in the *CCDC122-LACC1* gene, a frequency of 40% (*n* = 451) was demonstrated in the analysis of the population of Alagoas for the C allele, with 37% (*n* = 218) cases and 44% (*n* = 233) controls, indicating C as the polymorphic allele. The T allele presented a frequency of 63% (*n* = 372) in cases and 56% (*n* = 295) in controls. In the control group, the CC genotype presented a frequency of 22%, CT 44% and TT 34%. In cases, the CC genotype presented a frequency of 16%, CT 42% and TT 42%. Among the three genetic models analyzed in this case-control study (genotypes, allelic and carriers), the genotype was the most promising model to identify the differences between cases and controls in all populations. That said, a statistically significant association was observed in the “CC” genotype between the SNP *CCDC122-LACC1* and protection against leprosy in the population of Alagoas (OR_CC_ = 0.58, 95% CI = 0.36–0.93, *p* = 0.02, adjusted), a result that was replicated in the combined analysis of the populations of Northeastern Brazil (OR_CC_ = 0.65, 95% CI = 0.45–0.94, *p* = 0.02, adjusted). However, no association was identified in the isolated analysis of the populations of Pernambuco and Bahia (OR_CC_ = 0.79, 95% CI = 0.44–1.43, *p* = 0.44, adjusted). It was also possible to identify an association with a lower risk of leprosy in C carriers in the population of Alagoas (Table 1). The genotypic frequencies and genetic association for the *IL23R* SNP (rs3762318) in cases and controls are detailed in Table 1. When verifying the genetic models analyzed, no association was observed for this SNP in Alagoas, Pernambuco and Bahia, and in the combined analyses.

**Table 1:** Distribution of genotypic, allelic and allele carrier frequencies of the SNPs in the *CCDC122-LACC1* and *IL23R* gene in patients and controls and their association with leprosy per se

	Total *N* (%)	Cases *N* (%)	Controls *N* (%)	OR (CI 95%)	*p*	OR (CI 95%)*	p*
Northeastern Brazil (Alagoas/Pernambuco/Bahia)							
rs4942254/*CCDC122-LACC1*							
Genotypes/alleles	948/1,896	484/968	464/928				
**TT**	331 (35)	182 (38)	149 (32)	Reference			
CT	437 (46)	222 (46)	215(46)	0.85 (0.63–1.13)	0.25	0.85 (0.63–1.13)	0.25
CC	180 (19)	80 (17)	100 (22)	**0.65** **(0.45–0.94)**	**0.02**	**0.65** **(0.45–0.94)**	**0.02**
T	1099 (58)	586 (61)	513 (55)	Reference			
C	797 (42)	382 (39)	415 (45)	0.81 (0.62–1.04)	0.10	0.81 (0.62–1.04)	0.10
C carrier	617 (65)	302 (63)	315 (68)	0.78 (0.60–1.03)	0.08	0.78 (0.60–1.03)	0.08
rs3762318/*IL23R*							
Genotypes/alleles	946/1,892	483/966	463/926				
**GG**	31 (3)	18 (4)	13 (3)	Reference			
AG	284 (30)	145 (30)	139 (30)	0.75 (0.36–1.59)	0.46	0.75 (0.36–1.59)	0.46
AA	631 (67)	320 (66)	311 (67)	0.74 (0.36–1.54)	0.43	0.74 (0.36–1.54)	0.43
G	346 (18)	181 (19)	165 (18)	Reference			
A	1546 (82)	785 (81)	761 (82)	0.94 (0.68–1.31)	0.71	0.94 (0.68–1.31)	0.71
A carrier	915 (97)	465 (96)	450 (97)	0.75 (0.36–1.54)	0.43	0.75 (0.36–1.54)	0.43

*Results of OR (IC 95%) and *p* adjusted for sex. OR: Odds Ratio. CI: Confidence Interval. *p*: p-value. Genotypes and alleles in bold text were used as baseline

### Genetic association of SNPs according to operational classification

For the rs4942254 polymorphism (*CCDC122-LACC1*) in the analyses with the combined populations, a higher frequency of the T allele was observed in the PB (60%) and MB (61%) groups. The CT genotype was more frequent in MB (47%) than in PB (41%), while the CC genotype had the lowest frequency of 20% in PB and 16% in MB. In the models, no genetic associations were identified with the operational classification of leprosy in the populations of Alagoas, Pernambuco and Bahia, and in the combined populations. For the genetic SNP rs3762318 (*IL23R*), in the replication with the populations of Northeastern Brazil, a higher frequency of the A allele was observed in both PB (77%) and MB (82%). The AA genotype was more frequent in MB (67%) than in PB (61%), while the AG genotype had a frequency of 33% in PB and 29% in MB. Among the models analyzed, no genetic association was identified with any operational classification of leprosy in the study populations (Table 2).

**Table 2:** Distribution of genotypic, allelic and allele carrier frequencies of SNPs in the *CCDC122-LACC1* and *IL23R* genes in paucibacillary and multibacillary patients associated with leprosy

	Total *N* (%)	Multibacillary *N* (%)	Paucibacillary *N* (%)	OR (IC 95%)	*p*	OR (IC 95%)*	p*
Northeastern Brazil (Alagoas/Pernambuco/Bahia)							
rs4942254/*CCDC122-LACC1*							
Genotypes/alleles	484/968	418/836	66/132				
**TT**	182 (38)	156 (37)	26 (39)	Reference			
CT	222 (46)	195 (47)	27 (41)	1.20 (0.67–2.15)	0.53	1.08 (0.60–1.95)	0.80
CC	80 (17)	67 (16)	13 (20)	0.86 (0.42–1.77)	0.68	0.98 (0.46–2.05)	0.95
T	586 (61)	507 (61)	79 (60)	Reference			
C	382 (39)	329 (39)	53 (40)	0.97 (0.57–1.64)	0.90	1.00 (0.58–1.72)	0.99
C carrier	302 (63)	262 (63)	40 (61)	1.09 (0.64–1.86)	0.75	1.04 (0.61–1.80)	0.87
rs3762318/*IL23R*							
Genotypes/alleles	484/968	418/836	66/132				
**GG**	18 (4)	14 (3)	4 (6)	Reference			
AG	145 (30)	123 (29)	22 (33)	1.60 (0.48–5.30)	0.44	1.56 (0.46–5.36)	0.48
AA	320 (66)	280 (67)	40 (61)	2.00 (0.63–6.38)	0.24	1.87 (0.57–6.16)	0.30
G	181 (19)	151 (18)	30 (23)	Reference			
A	785 (81)	683 (82)	102 (77)	1.33 (0.71–2.49)	0.37	1.28 (0.67–2.42)	0.45
A carrier	465 (96)	403 (96)	62 (94)	1.86 (0.59–5.82)	0.29	1.76 (0.54–5.71)	0.34

*Results of OR (IC 95%) and *p* adjusted for sex. OR: Odds Ratio. CI: Confidence Interval. *p*: p-value. Genotypes and alleles in bold text were used as baseline

For the studied SNPs, the comparative groups of multibacillary patients versus controls were also performed. For the SNP rs4942254 (*CCDC122-LACC1*), it was possible to identify in the CC genotype a statistically significant association with a lower risk of leprosy in the population of Alagoas (OR_CC_ = 0.56, 95% CI = 0.34–0.92, *p* = 0.02), and in the combined analysis of the populations (OR_CC_ = 0.65, 95% CI = 0.45–0.94, *p* = 0.03). When analyzing the rs3762318 (*IL23R*) polymorphism, no statistically significant association was identified in any of the models analyzed, however, a suggestion of association with leprosy was identified in the AA genotype analyzed in the population of Alagoas individually (OR_AA_ = 0.34, 95% CI = 0.10–1.11, *p* = 0.07) (Table 3). Another comparative group also analyzed paucibacillary patients versus controls. For the genetic SNPs rs4942254 (*CCDC122-LACC1*) and rs3762318 (*IL23R*), no statistically significant associations were demonstrated in any of the models analyzed, in the study populations (Table 4).

**Table 3:** Distribution of genotypic, allelic and allele carrier frequencies of the SNPs in the *CCDC122-LACC1* and *IL23R* gene in patients and controls and their association with leprosy multibacillary

	Total *N* (%)	Multibacillary *N* (%)	Controls *N* (%)	OR (IC 95%)	*p*	OR (IC 95%)*	p*
Northeastern Brazil (Alagoas/Pernambuco/Bahia)							
rs4942254/*CCDC122-LACC1*							
Genotypes/alleles	877/1,754	413/826	464/928				
**TT**	302 (34)	153 (37)	149 (32)	Reference			
CT	408 (47)	193 (47)	215 (46)	0.87 (0.65–1.18)	0.38	0.87 (0.64–1.17)	0.35
CC	167 (19)	67 (16)	100 (22)	**0.65** **(0.45–0.96)**	**0.03**	**0.65** **(0.44–0.95)**	**0.03**
T	1012 (58)	499 (60)	513 (55)	Reference			
C	724 (42)	327 (40)	415 (45)	0.81 (0.62–1.06)	0.12	0.81 (0.62–1.05)	0.12
C carrier	575 (66)	260 (63)	315 (68)	0.80 (0.61–1.06)	0.13	0.80 (0.60–1.05)	0.11
rs3762318/*IL23R*							
Genotypes/alleles	877/1,754	413/826	463/926				
**GG**	27 (3)	14 (4)	13 (3)	Reference			
AG	262 (30)	123 (30)	139 (30)	0.82 (0.37–1.82)	0.63	0.83 (0.38–1.85)	0.65
AA	585 (66)	274 (67)	311 (67)	0.82 (0.38–1.77)	0.61	0.83 (0.38–1.85)	0.63
G	316 (18)	151 (18)	165 (18)	Reference			
A	1432 (82)	671 (81)	761 (82)	0.96 (0.68–1.36)	0.83	0.96 (0.68–1.36)	0.82
A carrier	915 (97)	465 (96)	450 (97)	0.82 (0.38–1.76)	0.61	0.82 (0.38–1.76)	0.61

*Results of OR (IC 95%) and *p* adjusted for sex. OR: Odds Ratio. CI: Confidence Interval. *p*: p-value. Genotypes and alleles in bold text were used as baseline

**Table 4:** Distribution of genotypic, allelic and allele carrier frequencies of the SNPs in the *CCDC122-LACC1* and *IL23R* gene in patients and controls and their association with leprosy paucibacillary

	Total *N* (%)	Paucibacillary *N* (%)	Controls *N* (%)			OR (IC 95%)	*p*	OR (IC 95%)*	p*
Northeastern Brazil (Alagoas/Pernambuco/Bahia)									
rs4942254/*CCDC122-LACC1*									
Genotypes/alleles	530/1,060	66/132	464/928						
**TT**	175 (33)	26 (39)	149 (32)			Reference			
CT	242 (46)	27 (41)	215 (46)			0.72 (0.40–1.28)	0.26	0.73 (0.41–1.31)	0.29
CC	113 (21)	13 (20)	100 (22)			0.74 (0.36–1.52)	0.42	0.83 (0.40–1.71)	0.61
T	592 (56)	79 (60)	513 (55)			Reference			
C	468 (44)	53 (40)	415 (45)			0.83 (0.49–1.40)	0.48	0.87 (0.51–1.49)	0.62
C carrier	355 (67)	40 (61)	315 (68)			0.73 (0.43–1.24)	0.24	0.76 (0.44–1.30)	0.32
rs3762318/*IL23R*									
Genotypes/alleles	529/1,058	66/132	463/928						
**GG**	17 (3)	4 (6)	13 (3)			Reference			
AG	161 (30)	22 (33)	139 (30)			0.51 (0.15–1.73)	0.28	0.44 (0.13–1.50)	0.19
AA	351 (66)	40 (61)	311 (67)			0.42 (013-1.34)	0.14	0.39 (0.12–1.30)	0.13
G	195 (18)	30 (23)	165 (18)			Reference			
A	863 (82)	102 (77)	761 (82)			0.74 (0.37–1.37)	0.34	0.78 (0.41–1.47)	0.44
A carrier	512 (96)	62 (94)	450 (97)			0.45 (0.14–1.42)	0.17	0.41 (0.12–1.33)	0.14

*Results of OR (IC 95%) and *p* adjusted for sex. OR: Odds Ratio. CI: Confidence Interval. *p*: p-value. Genotypes and alleles in bold text were used as baseline

## Discussion

This study evaluated the association of polymorphisms in the *CCDC122-LACC1* (rs4942254) and *IL23R* (rs3762318) genes with leprosy in two populations from Northeastern of Brazil. Among the patients involved in the population sample, 86.3% presented the multibacillary operational classification. The higher frequency of male patients (61.9%) was similar to data presented in epidemiological studies carried out in Alagoas, Amazonas, Minas Gerais, Rio de Janeiro and Pará, which indicated a greater presence of males and with an average age close to the present study [17, 27–30].

### The polymorphism in the *CCDC122-LACC1* gene and leprosy

The development and onset of leprosy are affected by the genetic aspects of the host [31]. Polymorphisms in the *CCDC122-LACC1* gene have been identified as being associated with inflammatory diseases such as leprosy, Crohn’s disease, among others [31, 34–39]. The association of the *CCDC122-LACC1* gene with leprosy has already been investigated in several different populations, such as Brazil, China, India, Mali, and Vietnam. India and Brazil have been the countries with the highest incidence rates of leprosy in the world over the last ten years [23]. However, this is the first study conducted in a population from Northeastern Brazilian. When verifying the results obtained regarding the association of SNP rs4942254 (*CCDC122-LACC1*) and leprosy, an association with protection against the development of the disease was identified in the comparison of the CC genotype in the population of Alagoas and this was replicated in the combined populations of Northeastern Brazil, which indicates similarity with another study that addressed the same polymorphism in other Brazilian populations [31]. For the comparison group MB versus controls, it was possible to identify an association with protection against leprosy in the CC homozygote in SNP rs4942254 (*CCDC122-LACC1*), in Alagoas and in the combined replication of populations.

When analyzing the available literature, similarity was found between the results obtained in the present study conducted with SNP rs4942254 (*CCDC122-LACC1*) and those previously. The frequency of the minority CC genotype in our study was 22% controls, similarly to the study from population of Southeastern Brazil [31], also with 22%. These similar findings can also be seen in the association analysis, which showed an association with protection against leprosy per se in the CC genotype (OR = 0.72, *p* = 0.003) [31], as demonstrated in this study (OR_CC_ = 0.58, *p* = 0.02, adjusted). In contrast, the study made out in the population of Northern Brazil [17], showed a frequency of the CC genotype of 13% controls. This difference between the findings in the present study was also seen in the analysis that showed no association with the disease (OR = 1.07, *p* = 0.602) [17].

In the Brazilian population, these three studies that investigated the role of the rs4942254 polymorphism (*CCDC122-LACC1*) represent these regions of Brazil: Northeastern (present study), Southeastern [31] and Northern [17]. When evaluating these studies, it is clear that the results were similar to those obtained in the population of the Southeastern region, which also showed an association with protection, and when comparing the Northern region (state of Amazonas), no association was found for the polymorphism studied.

Within the factors that could justify this discrepancy between the findings in the Brazilian population are possible peculiarities in the experimental design used and the different patterns of miscegenation in the populations investigated. Among these, there are also variations in genetic ancestry by Brazilian region. It is observed in the literature that the Northeastern region has a more expressive presence of African and European ancestry [32, 33]. While the Southeastern region has greater diversity and admixture of the three ancestry proportions (Native American, European and African, with greater proportions of the last two) [32]. These similarities in genetic characteristics between the two populations (Northeastern and Southeastern) may corroborate with the results observed in the present study. While comparing the difference in findings with the population of the Northern, which shows higher proportions of Native American ancestry [17, 32], this difference is due to the fact that the proportions of African and European genetic background are smaller compared to the Northeastern, which is reflected in the differences in associations that are identified [32, 33].

As for the other polymorphisms investigated in the same gene, an association with a lower risk of leprosy for the SNP rs9533634 (*CCDC122*) and susceptibility for the SNP rs3764147 (*LACC1*) was observed in the study carried out by Wong et al. [35, 36], which covered three populations (New Delhi, Calcutta and Mali) [36].

A study of families of leprosy patients, whose groups were divided by subtypes (286 MB descendants and 188 PB descendants), in Vietnam for the CCDC122-LACC1 *locus*, identified the polymorphic “A” alleles, “G” and “A” for SNPs rs3088362, rs3764147 and rs10507522, respectively [34]. These polymorphisms have been associated with susceptibility to leprosy, while the rs9533634 SNP has not been associated with the disease [34]. This study showed similarities with the genome-wide association study (GWAS) in Chinese patients [35], where only two SNPs were validated in the sample used, which was later also demonstrated in the study by Wong et al. [35, 36]. In an genetic association research carried out in the Chinese population, the SNPs studied (rs3088362, rs9533634, rs3764147 and rs10507522) were statistically significant associated with susceptibility to leprosy [37]. Another study with a Vietnamese population reestablished the susceptibility to leprosy of the SNV (single nucleotide variant) rs3764147 (p.lle254Val) (OR = 1.52, *p* = 5.06 × 10^−14^), polymorphic “G” allele, which has also been linked to an increased susceptibility to Crohn’s disease and other intestinal inflammation [38].

An association study in southwest China with the rs3764147 SNP, where genetic and expressional evidence points to *LACC1* as a leprosy susceptibility gene [39]. The present study demonstrated an association of the SNP rs4942254 (*CCDC122-LACC1*) with multibacillary leprosy in the population of Northeastern Brazil (OR_CC_ = 0.65, *p* = 0.03). When compared with the findings in the literature, we indicated the different SNPs of the same gene from a recent GWAS study with the same population using 17 different SNPs, initially suggesting that there is no significant association between PB and MB, later showed that the SNP rs3764147, polymorphic “A” allele (OR = 1.51, 95% CI = 1.26–1.81, *p* = < 0.0001) of the *LACC1* gene was strongly associated with susceptibility to leprosy, in addition to presenting a higher risk of tuberculoid and lepromatous leprosy, together with the presence of ENL-type reactional episodes, validating a status reactivity in this study [11].

### Polymorphisms in the *IL23R* gene and leprosy

The *IL23R* gene encodes the protein responsible for forming a receptor for the cytokine interleukin (IL)-23, and is part of the signaling pathway involving the gene product from another locus associated with leprosy, TNFSF15. With the β1 subunit of IL-12 (encoded by *IL12RB1*), *IL23R* participates in the IL-12, IL-23, and IFN-γ cascades, which have been reported to have essential roles in immunity to mycobacteria [21]. *IL23R* also plays a significant role in diseases such as: ankylosing spondylitis, Crohn’s disease, psoriatic arthritis, ulcerative colitis, Vogt-Koyanagi-Harada disease (VKH), and more recently, leprosy [16, 40–42].

In the present study, the frequencies of the AA genotype were observed to be 67% in population studied, and no statistically significant associations were found for the rs3762318 polymorphism in the *IL23R* gene with leprosy (OR_AA_ = 0.74, *p* = 0.43). When comparing these findings with those in the literature regarding the same genetic marker, studies show different results to those presented here. In the first study executed of this SNP, the association of the SNP in the *IL23R* gene with leprosy in the Chinese population, with a sample size of 3,301 patients and 5,299 individuals without the disease, in which the frequency of the AA genotype was 83%, and the analyses indicated an association with protection against leprosy (OR = 0.69, *p* = 3.27 × 10^− 11^) [18], as also demonstrated in the study carried out in southwest China (OR = 0.42, 95% CI = 0.26–0.68, *p* = 3.2E-04) [16].

This discrepancy between the association results between the two distinct populations, Brazil and China [16, 18], can be explained by some environmental and socioeconomic factors, such as nutrition and income, and genetic factors, such as bacterial genetic differences and host susceptibility. In Brazil, because it has a mixed-race population, where the Northeastern region has a greater expressiveness in its African and European genetics ancestry [32, 33, 35], these results may be different when performed on different populations, such as the Chinese population, where the proportions of ancestry are significantly lower than those of South American and African ancestries [43].

As observed in this study, it was not possible to identify an association between the SNP rs3762318 in the *IL23R* gene and the operational classifications of leprosy (OR_AA_ = 1.87, *p* = 0.30). Within compared with the findings in the literature, a study that analyzed the numbers of copy variations of the *IL23R* gene showed a significant increase in copies in PB patients (PB = 36.4%; Controls = 20.2%; *p* = 0.026), positively associating the gene to the PB form of leprosy [44]. These findings suggest an expansion of the understanding of the biological functions of *IL23R*, by discovering its involvement in susceptibility to infectious diseases that suggest involvement in autophagocytosis in the pathogenesis of leprosy [18].

Among the limitations faced during the conduct of the present study, the adherence to increasing the estimated sample size was mainly identified, which could lead to a possible bias in the study. Although this study prioritized the genetic aspects of leprosy, it is possible that other relevant variables were not fully explored due to methodological restrictions. However, the findings presented here remain consistent with the current literature and offer valuable insights for future discussions. Our study contributes to the expansion of the list of SNPs associated with leprosy in Brazil, in addition to providing for the first time the characterization of SNP association findings in the sampled population of Northeastern Brazil. This research provides knowledge on the genomic regions that influence the development of leprosy. Genetic studies contribute to understanding the molecular bases of susceptibility or protection to leprosy and the pathophysiology of the disease [17, 45]. The genetic data presented here will contribute to the development of a panel of genetic markers associated with leprosy. This panel can help predict individuals at greater risk of developing the disease or its more aggressive condition. This perspective implies new strategies to combat the disease, contributing to the creation of new public health policies.

## Conclusion

From the data presented, the SNP rs4942254 (*CCDC122-LACC1*) was associated with protection against leprosy, while the SNP rs3762318 (*IL23R*) was not associated with leprosy in populations from Northeastern Brazil. An association with the multibacillary operational classification of leprosy of the polymorphism in *CCDC122-LACC1* was also identified. It is expected that these results will contribute to expanding knowledge about the influence of SNP-type genetic markers on the immune response of leprosy patients, contributing to the understanding of the genetics and pathophysiology of the disease.

## Supplementary Information

Below is the link to the electronic supplementary material.

Supplementary Material 1

## Data Availability

The datasets used and/or analyzed during the current study are available from the corresponding author on reasonable request.

## Abbreviations

CCDC122: LACC1 Coiled-Coil Domain Containing 122-Laccase Domain Containing 1
IL23R: Interleukin-23 Receptor
PCR: Polymerase Chain Reaction
SNP: Single Nucleotide Polymorphism
NK: Natural Killer
FICF: Free and Informed Consent Form
HIV: Human Immunodeficiency Virus
PB: Paucibacillary
MB: Multibacillary
HWE: Hardy-Weinberg Equilibrium
OR: Odds Ratio
CI: Confidence Interval
WHO: World Health Organization
