# Supplementary material for: CCDC122-LACC1 gene polymorphism is associated with protection against leprosy in a population from Northeastern Brazil: a case-control study
Source: BMC Infect Dis. 2025 Dec 22;25:1792. doi: 10.1186/s12879-025-12391-3 (PMC12751134; doi:10.1186/s12879-025-12391-3)
Supplement: Supplementary file 1 — Supplementary Material 1 [file 12879_2025_12391_MOESM1_ESM.pdf]

Supplementary Table 1. Descriptive characteristics of the studied populations by sex, age and operational classification (WHO) for sampled total, cases and controls

	Total N (%)	Cases N (%)	Controls N (%)
Alagoas			
	562	298	264
Sex			
Male	348 (61.9)	180 (60.4)	168 (63.6)
Female	214 (38.1)	118 (39.6)	96 (36.4)
Age			
Mean (±SD)	48 (±16)	48 (±16)	33 (±11)
0-29	147 (26.2)	31 (10.4)	116 (43.9)
30-54	294 (52.3)	158 (53.0)	136 (51.5)
>55	121 (21.5)	109 (36.6)	12 (4.5)
Operational classification (WHO)			
PB		49 (16.4)	
MB	*	249 (83.6)	*
Pernambuco/Bahia			
	390	190	200
Sex			
Male	241 (61.8)	122 (64.2)	119 (59.5)
Female	149 (38.2)	68 (35.8)	81 (40.5)
Age			
Mean (±SD)	43 (±15)	51 (±14)	35 (±11)

0-29	95 (24.3)	20 (10.5)	75 (37.5)
30-54	195 (50.0)	90 (47.4)	113 (56.5)
>55	40 (10.2)	28 (14.7)	12 (6.0)
Operational classification (WHO)			
PB		18 (9.5)	
MB	*	172 (90.5)	*
Northeastern Brazil (Alagoas/Pernambuco/Bahia)	952	488	464
Sex			
Male	589 (61.9)	302 (61.9)	287 (61.9)
Female	363 (38.1)	186 (38.1)	177 (38.1)
Age			
Mean (±SD)	42 (±16)	49 (±15)	34 (±11)
0-29	242 (25.4)	51 (10.4)	191 (41.2)
30-54	497 (52.2)	248 (50.8)	249 (53.7)
>55	213 (22.4)	189 (38.7)	24 (5.2)
Operational classification (WHO)			
PB		67 (13.7)	
MB	*	421 (86.3)	*

SD: Standard Deviation; PB: Paucibacillary; MB: Multibacillary; *: Not applicable

Source: Research data, 2024.
